# Iron Deficiency Anemia at Time of Vaccination Predicts Decreased Vaccine Response and Iron Supplementation at Time of Vaccination Increases Humoral Vaccine Response: A Birth Cohort Study and a Randomized Trial Follow-Up Study in Kenyan Infants

**DOI:** 10.3389/fimmu.2020.01313

**Published:** 2020-07-13

**Authors:** Nicole U. Stoffel, Mary A. Uyoga, Francis M. Mutuku, Joe N. Frost, Edith Mwasi, Daniela Paganini, Fiona R. M. van der Klis, Indu J. Malhotra, A. Desiráe LaBeaud, Cristian Ricci, Simon Karanja, Hal Drakesmith, Charles H. King, Michael B. Zimmermann

**Affiliations:** ^1^Department of Health Sciences and Technology, Institute of Food, Nutrition and Health, Laboratory of Human Nutrition, ETH Zürich, Zurich, Switzerland; ^2^Department of Environment and Health Sciences, Technical University Mombasa, Mombasa, Kenya; ^3^MRC Human Immunology Unit, MRC Weatherall Institute of Molecular Medicine, John Radcliffe Hospital, Oxford, United Kingdom; ^4^Pediatrics Department, Msambweni County Referral Hospital, Msambweni, Kenya; ^5^Centre for Infectious Disease Control, National Institute for Public Health and Environment (RIVM), Bilthoven, Netherlands; ^6^Center for Global Health and Diseases, Case Western Reserve University School of Medicine, Cleveland, OH, United States; ^7^Division of Infectious Diseases, Department of Pediatrics, Lucille Packard Children's Hospital at Stanford School of Medicine, Stanford, CA, United States; ^8^Pediatric Epidemiology, Department of Pediatrics, Medical Faculty, Leipzig University, Leipzig, Germany; ^9^Department of Medical Epidemiology, College of Health Sciences, Jomo Kenyatta University of Agriculture and Technology, Nairobi, Kenya; ^10^Haematology Theme, NIHR Oxford Biomedical Research Centre, Oxford, United Kingdom

**Keywords:** iron deficiency, anemia, iron, vaccine response, seroconversion, infancy, Kenya

## Abstract

**Background:** Iron deficiency may impair adaptive immunity and is common among African infants at time of vaccination. Whether iron deficiency impairs vaccine response and whether iron supplementation improves humoral vaccine response is uncertain.

**Methods:** We performed two studies in southern coastal Kenya. In a birth cohort study, we followed infants to age 18 mo and assessed whether anemia or iron deficiency at time of vaccination predicted vaccine response to three-valent oral polio, diphtheria-tetanus-whole cell pertussis-*Haemophilus influenzae* type b vaccine, ten-valent pneumococcal-conjugate vaccine and measles vaccine. Primary outcomes were anti-vaccine-IgG and seroconversion at age 24 wk and 18 mo. In a randomized trial cohort follow-up, children received a micronutrient powder (MNP) with 5 mg iron daily or a MNP without iron for 4 mo starting at age 7.5 mo and received measles vaccine at 9 and 18 mo; primary outcomes were anti-measles IgG, seroconversion and avidity at age 11.5 mo and 4.5 y.

**Findings:** In the birth cohort study, 573 infants were enrolled and 303 completed the study. Controlling for sex, birthweight, anthropometric indices and maternal antibodies, hemoglobin at time of vaccination was the strongest positive predictor of: (A) anti-diphtheria and anti-pertussis-IgG at 24 wk (*p* = 0.0071, *p* = 0.0339) and 18 mo (*p* = 0.0182, *p* = 0.0360); (B) anti-pertussis filamentous hemagglutinin-IgG at 24 wk (*p* = 0.0423); and (C) anti-pneumococcus 19 IgG at 18 mo (*p* = 0.0129). Anemia and serum transferrin receptor at time of vaccination were the strongest predictors of seroconversion against diphtheria (*p* = 0.0484, *p* = 0.0439) and pneumococcus 19 at 18 mo (*p* = 0.0199, *p* = 0.0327). In the randomized trial, 155 infants were recruited, 127 and 88 were assessed at age 11.5 mo and 4.5 y. Compared to infants that did not receive iron, those who received iron at time of vaccination had higher anti-measles-IgG (*p* = 0.0415), seroconversion (*p* = 0.0531) and IgG avidity (*p* = 0.0425) at 11.5 mo.

**Interpretation:** In Kenyan infants, anemia and iron deficiency at time of vaccination predict decreased response to diphtheria, pertussis and pneumococcal vaccines. Primary response to measles vaccine may be increased by iron supplementation at time of vaccination. These findings argue that correction of iron deficiency during early infancy may improve vaccine response.

## Introduction

Increasing immunization efficacy and reducing anemia are key global pediatric health goals. Immunization programs have achieved high coverage, yet one in five children worldwide are not fully protected, contributing to 1.5 million child deaths annually from vaccine-preventable diseases (1). Vaccines often underperform in low- and middle-income countries (LMIC) (2, 3); for example, effectiveness of measles vaccine is generally <75% in Sub-Saharan Africa (4). Why vaccines underperform in LMIC remains uncertain (2, 3) but nutritional deficiencies may play a role (3, 5).

Over 40% of children <5 y worldwide are anemic, many due to iron deficiency (ID) (6). Anemia is particularly common in infants age <1 y in Sub-Saharan Africa: >70% of infants may be anemic at time of routine vaccination in the first year (7, 8). ID not only causes anemia, but also may impair adaptive immunity and thereby vaccine efficacy, although the data are equivocal (5, 9, 10). ID in mice attenuates T-cell dependent and independent antigen-specific antibody responses, and impairs cyclin E1 induction and S-phase entry during B-cell proliferation (10). Data from observational studies in humans are contradictory, in some, ID was associated with defective immune function, particularly T-cell immunity (5, 9). Clinically, a homozygous mutation in transferrin receptor (TfR)1 (the protein which transports iron into lymphocytes) causes severe combined immunodeficiency with low levels of circulating immunoglobulins (Ig) and decreased T and B cell proliferation *in vitro* (11). Data linking anemia, ID and vaccine response in LMIC are scarce; most data are from older studies that used outmoded methods to assess vaccine response (5).

In southern coastal Kenya, 70–75% of infants are anemic at the time they receive their routine vaccinations (8, 12). Therefore, we performed two studies to determine if anemia and/or ID during infancy affects vaccine response. In a birth cohort study, we followed infants to age 18 mo and assessed whether anemia or ID at time of vaccination predicted response to three-valent oral polio (OPV), diphtheria, tetanus, whole-cell pertussis, *Haemophilus influenzae* type b (Hib), ten-valent pneumococcal polysaccharide (PnPs) and measles vaccines (MV). Primary outcomes were anti-vaccine serum IgG and seroconversion at age 24 wk (primary response) and 18 mo (secondary response). We hypothesized that anemia and/or ID at time of vaccination would predict lower serum IgG and reduced seroconversion at these ages. In a randomized trial cohort follow-up, children received a micronutrient powder (MNP) with 5 mg iron daily or a MNP without iron for 4 mo starting at age 7.5 mo and received MV at age 9 and 18 mo. Primary outcomes were anti-measles serum IgG, seroconversion and IgG avidity assessed at 11.5 mo (primary response) and 4.5 y (secondary response). We hypothesized that iron supplementation at time of first MV would result in higher anti-measles serum IgG, seroconversion and IgG avidity at both time points.

## Participants and Methods

These studies were done in Kwale County, in southern coastal Kenya. We recruited mothers and infants from the maternal care and infant growth monitoring and vaccination clinics in Msambweni County Referral Hospital and surrounding health centers. The two studies enrolled independent groups of infants. For the birth cohort study, we obtained ethical approval from the institutional review boards at Kenyatta National Hospital/University of Nairobi (KNH/UoN), Case Western Reserve University and the Stanford University School of Medicine. For the randomized trial, we obtained ethical approval from the institutional review boards of the KNH/UoN and the Zurich Cantonal Ethical Commission; approval for the follow-up study was given by KNH/UoN. The randomized trial was registered and updated on clinical trials (NCT02118402). Caregivers gave informed consent by either a written signature or a fingerprint.

### Study Design

**Figure 1:**
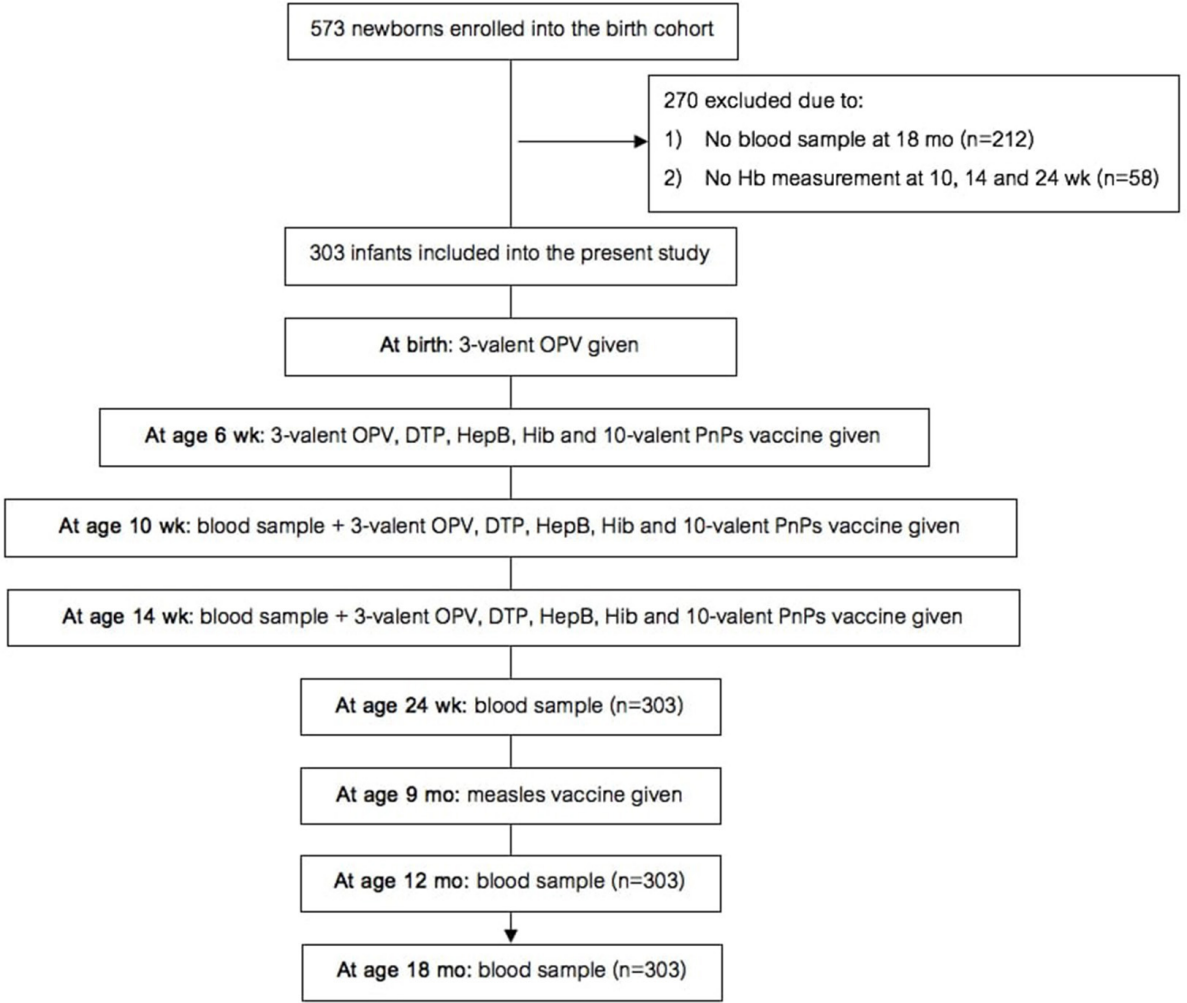
Design of the birth cohort study. Hb, hemoglobin; OPV, oral polio vaccine; DTP, diphtheria-tetanus-pertussis; HepB, hepatitis B; Hib, *Haemophilus influenzae* type b; PnPs, pneumococcal polysaccharide.

The birth cohort study (study design in Figure 1) was nested within a larger longitudinal maternal-child cohort study on links between maternal parasitic infections and infant vaccine response. In the original maternal-child cohort study [details in (13)], healthy pregnant women ≥15 y of age in the 2nd or 3rd trimester of pregnancy were invited to participate in the study when they presented for antenatal care. If they had an uncomplicated delivery of a healthy infant (at ≥34 wk with a birthweight ≥1500 g and an APGAR score ≥5), then the infant was enrolled into this study. Additional inclusion criteria for this cohort study were: (1) infant remained in the study and provided samples for anti-vaccine serum IgG concentrations at age 18 mo; and (2) one or more hemoglobin (Hb) measurement(s) were done at age 10, 14, or 24 wk.

At delivery, we collected a whole blood sample from the umbilical vein. We obtained whole blood samples from the infants at age 10 and 14 wk by heel prick, and at age 24 wk, 12 and 18 mo by venipuncture, and trained staff measured length and weight. Under supervision of the study team, all infants received standardized immunizations following Kenyan Ministry of Health guidelines, as follows: (A) three-valent OPV with serotypes 1, 2, 3 at birth; (B) at age 6, 10, and 14 wk, the infants were given OPV, diphtheria-tetanus-whole cell pertussis-hepatitis B-Hib vaccine, ten-valent PnPs vaccine (Synflorix) with serotypes 1, 4, 5, 6B, 7F, 9V, 14, 18C, 19F, and 23F; and (C) at age 9 mo, they received the monovalent MV. At age 18 mo, we collected a blood sample before the second MV was given. All infants in the study received vitamin A supplementation every 6 months starting at age 24 wk as per Kenyan guidelines. At each visit, trained staff asked the caregiver if the infant had received malaria treatment since the last visit.

**Figure 2:**
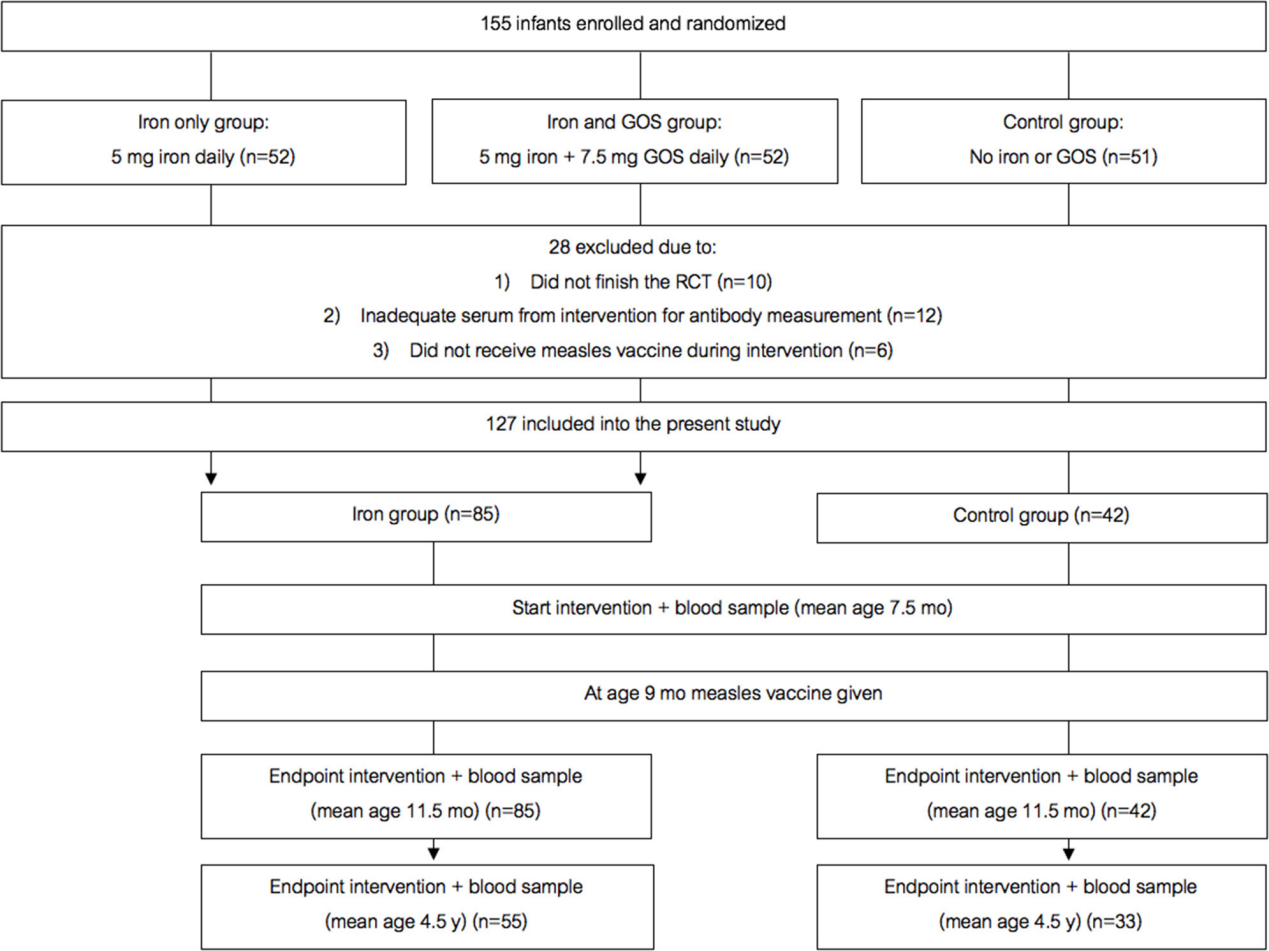
Design of the randomized controlled trial follow-up study. GOS, galacto-oligosaccharides.

Figure 2 shows the study design for the randomized trial cohort follow-up study. In a double-blind, controlled trial [details in (12)], we randomized 155 Kenyan infants (mean age 7.5 mo) to receive daily for 4 mo: (A) a MNP without iron (control); (2) the identical MNP but containing 5 mg of iron as ferrous fumarate and sodium iron ethylenediaminetetraacetic acid (NaFeEDTA) (iron only group); or (3) the identical MNP as the iron only group but with 7.5 g galacto-oligosaccharides (GOS) (Vivinal GOS, Friesland Campina, Wageningen, The Netherlands) (iron and GOS group). 145 infants completed the intervention trial. Out of these 145 infants, all except six received a monovalent MV at age 9 mo (during the intervention). About 3.5 years later, we contacted all families with children who were in the original trial, with the exception of the six children who had received the MV before they entered the intervention trial, and 12 children for whom we did not have adequate plasma to measure the MV response; thus, 127 children were included in the present trial. From these 127 children, 88 families provided consent for the follow-up measurements; of these, 93% of children had received a second dose of MV at age 18 mo as recorded in their vaccination booklets. We measured child weight and length/height and collected a venous blood sample at age 7.5 mo, before they received the first MV, at endpoint of the intervention (at age 11.5 mo), and at follow up (at age 4.5 y).

### Laboratory Analysis

We measured Hb on the day of collection in both studies using a Hemocue spectrophotometer (Hemocue, Ångelholm, Sweden), with the exception of the 24 wk, 12 and 18 mo visits in the birth cohort study, when we measured Hb using an Automated Hematology Analyzer (Nihon Kohden model: 82 MEK 7222). Serum was separated by centrifugation and frozen on the day of collection until analysis. We measured serum TfR, serum ferritin (SF), high-sensitive C-reactive protein (CRP) and α-1 glycoprotein (AGP) by using a combined sandwich enzyme-linked immunosorbent assay (14). Serum IgG concentrations against PnPs (ten serotypes: 1, 4, 5, 6B, 7F, 9V, 14, 18C, 19F, 23F), purified polyribosylribitol phosphate (PRP) Hib, and diphtheria CRM197 toxoid antigens were measured at Stanford University, Palo Alto, CA, USA using a fluorescent multiplexed bead-based immunoassay (Luminex, Austin TX) (13). The twelve vaccine antigens were coupled to carboxylated microspheres, incubated with patient serum and quantified on a BioPlex MAGPIX multiplex reader (BioRad, Hercules, CA) (13). Serum IgG against tetanus toxoid, pertussis (pertussis toxin, filamentous hemagglutinin (FHA), and pertactin) measles and polio, and avidity of measles IgG, were measured at the National Institute for Public Health and the Environment, Bilthoven, Netherlands. Serum IgG against tetanus, pertussis and measles were simultaneously measured using a multiplex immunoassay (15, 16). Serum IgG against polio was measured using a multiplex poliovirus binding inhibition assay (17). Measles IgG avidity was determined by modifying a bead-based serum IgG immunoassay against measles. After incubation of measles virus-conjugated fluorescent beads with serum and before labeling, beads were washed with phosphate-buffered saline and subsequently left untreated or treated for 10 min with 1.5 M ammonium thiocyanate [NH_4_SCN], as described previously (18). The avidity index was defined as the percentage of antibodies that remain bound to the beads after treatment with thiocyanate, calculated as: [amount of IgG after thiocyanate treatment]/[amount of IgG without treatment] × 100 (18).

### Statistical Analysis

We conducted statistical analyses with SPSS (IBM SPSS statistics, Version 24). Data were checked for normality by Shapiro-Wilk tests and by visual inspection of histogram plots. Non-normally distributed data were logarithmically (log10) transformed for statistical analyses. Descriptive data were expressed as mean ± SD (for normally distributed data) or median (IQR) for non-normally distributed data. We defined ID at 24 wk as either SF <12 μg/L or 2) TfR >8.3 μg/ml, and at 12 and 18 mo as SF <12 μg/L (14) because we did not measure TfR at these later ages. We did not define ID at earlier time points because consensus cut-offs are unavailable for ages <24 wk. We defined anemia as Hb <10.5 g/dl at age 10 and 14 wk, based on proposed cut-off values for Hb at age 16 wk in iron-replete infants (19) and as Hb <11.0 g/dl at age 24 wk, 12 and 18 mo (20). We defined mild, moderate and severe anemia at 10 and 14 wk as an Hb of 9.5–10.5 g/dl, 7.0–9.4 g/dl and <7.0 g/dl, respectively; and at 24 wk, 12 and 18 mo as an Hb of 10.0–11.0 g/dl, 7.0–9.9 g/dl and <7.0 g/dl, respectively (20). We defined ID anemia as anemia with ID based on the above cut-offs. At all ages, we defined CRP and AGP >5 mg/L and >1 g/l respectively as elevated, indicating inflammation (14). Vitamin A deficiency was defined as RBP <0.7 μmol/L at 24 wk (14). We defined immunological response as protective when participants had anti-diphtheria serum IgG concentrations ≥0.1 IU/ml (21), anti-tetanus IgG >0.5 IU/ml (22), anti-Hib IgG >1.0 μg/ml (22), anti-PnPs IgG ≥0.35 μg/ml (23), anti-measles IgG ≥0.12 IU/ml (24) and anti-OPV IgG ≥0.6, ≥2.1 and ≥0.7 IU/ml for S1, 2, and 3 (25). There is no protective serum concentration established for anti-pertussis IgG (22). We converted infant anthropometric measurements to age-adjusted z-scores in Anthro software; children with a length/height-for-age z-score < -2 were categorized as stunted; those with a weight-for-age z-score < -2 as underweight and those with a weight-for-height z-score < -2 as wasted. For the birth cohort study, we defined primary and secondary vaccine response as anti-vaccine serum IgG concentrations and seroconversion at 10 wk and 14.5 mo after vaccination, respectively. For the randomized trial, we defined primary and secondary vaccine response as anti-vaccine serum IgG concentrations and seroconversion at 4 wk and 3.5 y after vaccination, respectively.

In the birth cohort study, we used linear regression analyses on anti-vaccine serum IgG concentrations at age 24 wk (primary vaccine response) and at 18 mo (secondary response) to assess the effect of anemia and ID at the time of vaccination on vaccine response. For anthropometrics and Hb, we used the mean value at age 10, 14, and 24 wk to represent that variable at time of vaccination. In addition to mean Hb, we sequentially added known potential predictors of vaccine response: sex, birthweight, mean anthropometrics, and maternal serum IgG concentrations (in cord blood and at age 10 wk) to the models. We performed logistic regression on seroconversion against diphtheria, tetanus, Hib, PnPs and OPV at age 24 wk and 18 mo, and MV at 12 and 18 mo, including the above covariates except Hb, and added moderate-severe anemia (yes/no) as a discrete variable to the models. We repeated the above analyses including TfR (as an indicator of iron-deficient erythropoiesis), RBP and AGP at age 24 wk. In all regression models we did bootstrapping with a resampling size of 1000.

In the randomized trial, we used a random intercept linear mixed effect model (LMM) analysis with Bonferroni corrected multiple comparisons to assess the effect of group (iron or control) at the time of MV. We performed LMM with anti-measles serum IgG concentrations as the dependent variable in two models to assess primary and secondary vaccine response. In the first model, we included anti-measles serum IgG measured at age 7.5 and 11.5 mo; in the second model, we included anti-measles serum IgG measured at age 7.5 mo and 4.5 y. We sequentially added covariates to the models that are potential predictors of vaccine response: age, weight-for-height z-score (WHZ), IDA and RBP. By adding age to the models, we corrected for differences between subjects in time elapsed from baseline to vaccination and from vaccination to endpoint of the intervention. Controlling for the same covariates, we performed logistic regression on seroconversion and antibody avidity at age 11.5 mo and 4.5 y. To create a binary variable for high and low avidity, we divided the avidity distribution in half, and described the upper half as high avidity. For both, we used the covariates measured at 11.5 mo (reflecting intervention and time of vaccination).

## Results

### Birth Cohort Study

The birth cohort study began recruiting in August 2013 and follow up was to May 2017. At birth, we enrolled 573 newborns into the study and collected a cord blood sample. At age 18 mo, 361 infants remained in the study and provided samples. 58 of these infants did not have one or more Hb measurement(s) at age 10, 14, or 24 wk. Thus, 303 infants (129 male, 174 female) were included in the data analysis (Figure 1). Only ten infants reportedly received malaria treatment at age 0–24 wk. Mean ±SD birthweight was 3,003 ± 457,g and 34 infants were low birthweight (LBW) (<2,500 g); based on the inclusion criteria, none of the infants were preterm. All infants received all vaccines at 6, 10 and 14 wk; 99% of the infants received the MV at 9 mo.

### Nutritional Status, Anemia, and Iron Deficiency

**Figure 3:**
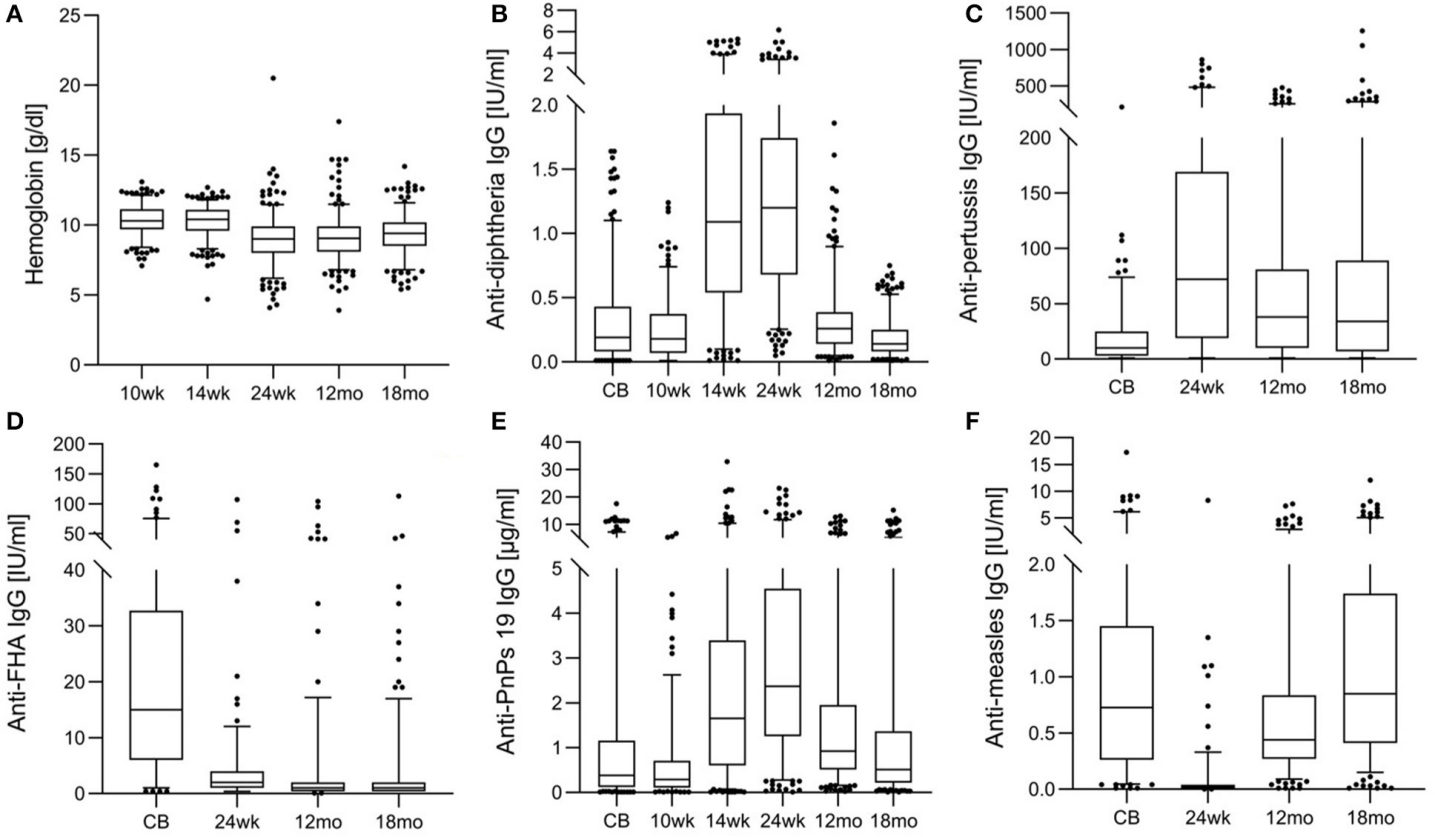
Hemoglobin and anti-vaccine serum IgG concentrations from birth until 18 months in the birth cohort of Kenyan infants. **(A)** Hemoglobin, **(B)** anti-diphtheria IgG, **(C)** anti-pertussis toxin IgG, **(D)** anti- filamentous hemagglutinin (FHA) IgG, **(E)** anti-pneumococcus serotype 19 (PnPs 19) IgG, and **(F)** anti-measles IgG.

Table 1 shows anthropometrics, iron and inflammation status from age 10 wk to 18 mo. At 24 wk, the prevalence of stunting and underweight were 29.0 and 10.0%, suggesting that overall, the infants were mild-to-moderately malnourished, but the prevalence of wasting was only 3.5%. At age 10 and 14 wk, just over half of infants were anemic; however, median SF was high and median TfR was in the normal range. At age 24 wk and 12 mo, median Hb was only 9.0 g/dl (Figure 3A) and >90% of infants were anemic: nearly all the anemia was moderate or severe; low median SF and high median TfR suggested iron depletion in the majority of infants (Table 1). At 24 wk, the prevalence of vitamin A deficiency was 47%, and 37% of infants showed signs of inflammation, likely due to common infections.

**Table 1:** Age, anthropometrics, biomarkers of iron, vitamin A and inflammation, and prevalence of iron deficiency, anemia, iron deficiency anemia, vitamin A deficiency and inflammation in the birth cohort study of Kenyan infants.

	**10 weeks**	**14 weeks**	**24 weeks**	**12 months**	**18 months**
Age, wk or mo	10.4 (10.4 − 10.8) (*n* = 262)	14.8 (14.4 − 15.2) (*n* = 287)	27.6 (26.2 − 28.4) (*n* = 285)	13.5 (13.1 − 14.4) (*n* = 285)	19.9 (19.5 − 20.8) (*n* = 289)
Height-for-age z-score	−1.37 (−2.52 −0.37) (*n* = 260)	−1.52 (−2.63 − 0.48) (*n* = 280)	−1.36 (−2.22 − 0.49) (*n* = 279)	−1.72 (−2.44 −1.04) (*n* = 280)	−1.87 (−2.63 −1.22) (*n* = 282)
Stunting, *n* (%)	99 (38.1)	102 (36.4)	81 (29.0)	115 (41.1)	125 (44.3)
Weight-for-age z-score	−0.55 (−1.27 − 0.03) (*n* = 260)	−0.56 (−1.34 −0.10) (*n* = 280)	−0.72 (−1.38 − 0.03) (*n* = 279)	−0.97 (−1.51 − 0.30) (*n* = 280)	−1.09 (−1.67 − 0.51) (*n* = 283)
Underweight, *n* (%)	31 (11.9)	35 (12.5)	28 (10.0)	37 (13.2)	46 (16.3)
Weight-for-height z-score	0.85 (−0.15 − 1.83) (*n* = 262)	0.78 (−0.37 − 1.83) (*n* = 282)	0.16 (−0.54 − 1.09) (*n* = 286)	−0.13 (−0.80 − 0.53) (*n* = 282)	−0.13 (−0.96 − 0.55) (*n* = 291)
Wasting, *n* (%)	8 (3.1)	8 (2.8)	10 (3.5)	12 (4.3)	16 (5.5)
Hemoglobin, g/dl	10.3 (9.7 − 11.1) (*n* = 254)	10.4 (9.6 − 11.1) (*n* = 284)	9.0 (8.0 − 9.9) (*n* = 283)	9.0 (8.1 − 9.9) (*n* = 282)	9.4 (8.5 − 10.3) (*n* = 293)
Serum ferritin, μg/L	171.6 (134.2 − 189.8) (*n* = 90)	153.5 (126.8 − 180.8) (*n* = 127)	23.4 (12.4 − 64.9) (*n* = 160)	12.0 (7.0 − 35.7) (*n* = 145)	12.6 (6.2 − 32.4) (*n* = 137)
Serum transferrin receptor, mg/L	4.1 (3.0 − 6.0) (*n* = 90)	4.6 (3.2 − 6.7) (*n* = 127)	8.3 (6.8 − 10.6) (*n* = 160)	-	-
Anemia, *n* (%)	136 (53.5)	149 (52.5)	263 (92.9)	263 (93.3)	258 (88.1)
Mild anemia, *n* (%)	84 (33.0)	84 (29.6)	44 (15.5)	48 (17.0)	58 (19.8)
Moderate anemia, *n* (%)	52 (20.5)	64 (22.5)	187 (66.1)	199 (70.6)	180 (61.4)
Severe anemia, n (%)	-	1 (0.4)	32 (11.3)	16 (5.7)	20 (6.8)
Iron deficiency, *n* (%)	-	-	92 (57.5)	72 (49.7)	64 (47.1)
Iron deficiency anemia, *n* (%)	-	-	87 (56.9)	70 (48.6)	57 (41.9)
Retinol binding protein, μmol/L	0.37 (0.28 − 0.52) (*n* = 90)	0.36 (0.28 − 0.50) (*n* = 127)	0.74 (0.56 − 0.89) (*n* = 160)	-	-
Vitamin A deficiency, *n* (%)			75 (46.9)	-	-
α-glycoprotein, g/L	0.23 (0.14 − 0.33) (*n* = 90)	0.24 (0.15 − 0.39) (*n* = 127)	0.71 (0.45 − 1.21) (*n* = 160)	-	-
C-reactive protein, mg/L	0.24 (0.13 − 0.89) (*n* = 90)	0.30 (0.18 − 0.92) (*n* = 127)	1.0 (0.28 − 5.42) (*n* = 160)	0.92 (0.21 − 3.68) (*n* = 146)	0.78 (0.22 − 2.94) (*n* = 132)
Inflammation, *n* (%)	10 (11.1)	8 (6.3)	59 (36.9)	31 (21.2)	22 (16.7)

*Data as medians (IQR). Definitions of stunting, underweight, wasting, anemia, iron deficiency, iron deficiency anemia, vitamin A deficiency and inflammation are given in the text*.

### Anti-vaccine Serum IgG Concentrations and Seroconversion

Table 2 shows anti-vaccine serum IgG concentrations and seroconversion for the measured vaccines. At age 24 wk, the primary vaccine response against diphtheria and tetanus was high, with only 1 and 14% of infants seronegative, while response to Hib was lower, with 45% seronegative. The primary response to the PnPs serotypes varied, the highest response was to PnPs 7 and PnPs 19, with only 3 and 6% seronegative at 24 wk, while the lowest response was to PnPs 1 and PnPs 23, with 26 and 24% of infants seronegative at 24 wk. The highest anti-vaccine serum IgG concentrations against many antigens in the diphtheria-tetanus-whole cell pertussis- Hib vaccine were present at 24 wk, and serum IgG waned at 12 and 18 mo (Figures 3B–E). At 18 mo, the secondary vaccine response varied: 16, 32, 62, and 68% were seronegative against OPV3, diphtheria, tetanus and Hib. Secondary response to the PnPs serotypes varied widely: the lowest response was against PnPs 5 (80% seronegative), while the highest response was against PnPs 6 (24% seronegative). MV response was high at 12 and 18 mo, before the second vaccination, with only 7 and 4% seronegative (Table 2 and Figure 3F). Notably, in cord blood, serum IgG against tetanus and measles were high, suggesting high transfer of maternal antibodies.

**Table 2:** Anti-vaccine serum IgG concentrations and seroconversion for diphtheria, tetanus, pertussis, *Haemophilus influenza b*, ten pneumococcus serotypes, measles and three oral polio serotypes in the birth cohort of Kenyan infants.

	**Cord blood**	**10 weeks**	**14 weeks**	**24 weeks**	**12 months**	**18 months**
Diphtheria, IU/ml	0.20 (0.08–0.43) (*n* = 275)	0.18 (0.07–0.37) (*n* = 218)	1.10 (0.54–1.94) (*n* = 258)	1.20 (0.68–1.75) (*n* = 262)	0.26 (0.14–0.39) (*n* = 260)	0.14 (0.08–0.25) (*n* = 303)
Seronegative, *n* (%)	77 (28.0)	71 (32.6)	11 (4.3)	3 (1.1)	37 (14.2)	97 (32.0)
Tetanus, IU/ml	2.74 (0.94–6.68) (*n* = 168)	NA	NA	1.28 (0.68–2.37) (*n* = 179)	0.51 (0.29–0.93) (*n* = 213)	0.40 (0.21–0.72) (*n* = 229)
Seronegative, *n* (%)	25 (14.9)			25 (14.0)	101 (47.4)	142 (62.0)
Pertussis toxin, IU/ml	10 (3-25) (*n* = 168)	NA	NA	72 (17–179) (*n* = 179)	38 (10-81) (*n* = 213)	34 (7-89) (*n* = 229)
Pertussis, Filamentous hemagglutinin, IU/ml	15 (6-33) (*n* = 168)	NA	NA	2 (1–4) (*n* = 179)	1 (0.4–2) (*n* = 213)	1 (0.4–2) (*n* = 229)
Pertussis, Pertactin, IU/ml	3.0 (1.0–8.0) (*n* = 168)	NA	NA	19.0 (7.0–35.0) (*n* = 179)	5.0 (2.0–10.0) (*n* = 213)	4.0 (2.0–8.0) (*n* = 229)
*Haemophilus influenzae* b, μg/ml	0.06 (0.02–0.26) (*n* = 283)	0.08 (0.01–0.17) (*n* = 220)	0.28 (0.08–0.85) (*n* = 258)	1.07 (0.43–2.34) (*n* = 268)	0.65 (0.27–1.60) (*n* = 260)	0.54 (0.15–1.25) (*n* = 297)
Seronegative, *n* (%)	267 (94.3)	215 (97.7)	199 (77.1)	121 (45.1)	160 (61.5)	201 (67.7)
Pneumococcus 1, μg/ml	0.01 (0.00–0.04) (*n* = 286)	0.09 (0.04–0.37) (*n* = 218)	0.48 (0.21–0.96) (*n* = 253)	0.70 (0.33–1.36) (*n* = 266)	0.29 (0.14–0.59) (*n* = 259)	0.18 (0.09–0.36) (*n* = 296)
Seronegative, *n* (%)	279 (97.6)	163 (74.8)	92 (36.4)	70 (26.3)	151 (58.3)	221 (74.7)
Pneumococcus 4, μg/ml	0.04 (0.02–0.09) (*n* = 278)	0.05 (0.03–0.15) (*n* = 218)	0.31 (0.13–0.85) (*n* = 259)	0.87 (0.45–1.43) (*n* = 265)	0.42 (0.22–0.75) (*n* = 258)	0.24 (0.12–0.45) (*n* = 298)
Seronegative, *n* (%)	247 (88.8)	194 (89.0)	138 (53.3)	46 (17.4)	108 (41.9)	194 (65.1)
Pneumococcus 5, μg/ml	0.02 (0.01–0.06) (*n* = 286)	0.09 (0.03–0.30) (*n* = 217)	0.59 (0.23–1.21) (*n* = 257)	0.70 (0.38–1.29) (*n* = 271)	0.27 (0.14–0.50) (*n* = 263)	0.17 (0.08–0.30) (*n* = 298)
Seronegative, *n* (%)	280 (97.9)	169 (77.9)	89 (34.6)	58 (21.4)	158 (60.1)	239 (80.2)
Pneumococcus 6, μg/ml	0.06 (0.02–0.27) (*n* = 282)	0.01 (0.00–0.04) (*n* = 219)	0.10 (0.02–0.39) (*n* = 255)	2.05 (0.87–3.75) (*n* = 267)	1.56 (0.68–3.15) (*n* = 261)	0.78 (0.36–1.61) (*n* = 294)
Seronegative, *n* (%)	225 (79.8)	200 (91.3)	186 (72.9)	36 (13.5)	32 (12.3)	69 (23.5)
Pneumococcus 7, μg/ml	0.05 (0.01–0.25) (*n* = 286)	0.11 (0.04–0.34) (*n* = 219)	0.71 (0.30–1.85) (*n* = 259)	1.66 (1.01–3.05) (*n* = 267)	0.86 (0.42–1.65) (*n* = 261)	0.42 (0.20–0.81) (*n* = 297)
Seronegative, *n* (%)	221 (77.3)	165 (75.3)	75 (29.0)	8 (3.0)	47 (18.0)	133 (44.8)
Pneumococcus 9, μg/ml	0.07 (0.02–0.22) (*n* = 286)	0.04 (0.02–0.14) (*n* = 221)	0.71 (0.26–1.62) (*n* = 259)	1.04 (0.61–1.87) (*n* = 267)	0.62 (0.35–1.15) (*n* = 262)	0.36 (0.19–0.78) (*n* = 298)
Seronegative, *n* (%)	241 (84.3)	194 (87.8)	78 (30.1)	22 (8.2)	64 (24.4)	139 (46.6)
Pneumococcus 14, μg/ml	1.64 (0.44–3.60) (*n* = 283)	0.30 (0.11–0.94) (*n* = 219)	0.97 (0.42–2.41) (*n* = 259)	1.94 (0.88–3.66) (*n* = 268)	0.55 (0.22–1.12) (*n* = 259)	0.33 (0.12–0.77) (*n* = 294)
Seronegative, *n* (%)	59 (20.8)	114 (52.1)	56 (21.6)	29 (10.8)	87 (33.6)	154 (52.4)
Pneumococcus 18, μg/ml	0.09 (0.28–0.30) (*n* = 284)	0.05 (0.02–0.15) (*n* = 220)	0.66 (0.21–1.92) (*n* = 256)	1.84 (0.82–5.45) (*n* = 268)	0.60 (0.30–1.04) (*n* = 263)	0.39 (0.18–0.69) (*n* = 299)
Seronegative, *n* (%)	222 (78.2)	192 (87.3)	89 (34.8)	20 (7.5)	76 (28.9)	133 (44.5)
Pneumococcus 19, μg/ml	0.38 (0.12–1.16) (*n* = 280)	0.29 (0.11–0.70) (*n* = 217)	1.65 (0.60–3.39) (*n* = 255)	2.37 (1.25–4.55) (*n* = 269)	0.92 (0.51–1.95) (*n* = 263)	0.51 (0.22–1.36) (*n* = 298)
Seronegative, *n* (%)	130 (46.4)	122 (56.2)	43 (16.9)	16 (5.9)	36 (13.7)	115 (38.6)
Pneumococcus 23, μg/ml	0.10 (0.03–0.40) (*n* = 281)	0.06 (0.02–0.13) (*n* = 221)	0.24 (0.11–0.65) (*n* = 256)	0.80 (0.35–1.38) (*n* = 271)	0.40 (0.21–0.85) (*n* = 264)	0.23 (0.09–0.45) (*n* = 298)
Seronegative, *n* (%)	200 (71.2)	202 (91.4)	154 (60.2)	64 (23.6)	113 (42.8)	198 (66.4)
Measles, IU/ml	0.73 (0.26–1.45) (*n* = 168)	NA	NA	0.02 (0.01–0.04) (*n* = 179)	0.44 (0.27–0.84) (*n* = 213)	0.85 (0.41–1.74) (*n* = 229)
Seronegative, *n* (%)	19 (11.3)			161 (89.9)	14 (6.6)	10 (4.4)
Poliomyelitis 1, IU/ml	0.20 (0.10–0.72) (*n* = 167)	NA	NA	6.84 (3.70–15.19) (*n* = 177)	5.34 (2.57–8.74) (*n* = 208)	4.13 (1.99–8.11) (*n* = 227)
Seronegative, *n* (%)	121 (72.5)			5 (2.8)	5 (2.4)	10 (4.4)
Poliomyelitis 2, IU/ml	1.38 (0.72–3.51) (*n* = 167)	NA	NA	21.54 (11.80–33.79) (*n* = 177)	14.48 (7.70–25.71) (*n* = 209)	14.94 (6.62–30.82) (*n* = 227)
Seronegative, *n* (%)	103 (61.7)			5 (2.8)	10 (4.8)	14 (6.2)
Poliomyelitis 3, IU/ml	0.19 (0.10–0.34) (*n* = 167)	NA	NA	2.84 (1.23–4.84) (*n* = 177)	2.70 (1.26–5.12) (*n* = 209)	1.73 (0.96–4.47) (*n* = 228)
Seronegative, *n* (%)	149 (89.2)			27 (15.3)	20 (9.6)	37 (16.2)

*Data as medians (IQR). NA, not available. Definitions of seroconversion against the different vaccines are given in the text*.

### Predictors of Anti-diphtheria Serum IgG Concentrations and Seroconversion

Table 3 shows the associations of Hb and moderate to severe anemia, and vaccine response. For diphtheria, controlling for sex, birthweight, anthropometrics and maternal antibodies, Hb at time of vaccination was the strongest positive predictor of anti-vaccine serum IgG at age 24 wk (*p* = 0.0071) and 18 mo (*p* = 0.0182). Moderate or severe anemia was the strongest risk factor for seronegativity at age 18 mo: anemic infants had 2.1 times the risk of being unprotected against diphtheria at 18 mo (*p* = 0.0484) (Table 3). Table 4 shows the associations of TfR (as an indicator of iron-deficient erythropoiesis) and vaccine response. Controlling for sex, birthweight, anthropometrics, inflammation, vitamin A status and maternal antibodies, TfR at age 24 wk was the strongest risk factor for seronegativity against diphtheria at 18 mo (*p* = 0.0334).

**Table 3:** Associations between hemoglobin (Hb) and moderate to severe anemia (Hb <9.5 g/dl), and anti-diphtheria, anti-pertussis, anti-pneumococcus serotype 19, and anti-measles serum IgG concentrations and/or seroconversion, in the birth cohort of Kenyan infants.

**DIPHTHERIA**			
	**B**	**Standard error of B**	**Standardized β**
**Anti-diphtheria IgG at 24 weeks:** ***R***^**2**^ **=** **0.087**			
Sex	0.036	0.056	0.052
Birthweight	−0.505	0.441	−0.099
Anti-diphtheria IgG in cord blood	0.048	0.051	0.073
Anti-diphtheria IgG at age 10 wk	0.091	0.052	0.139
Height-for-age z-score (mean at 10 to 24 wk)	−0.014	0.023	−0.053
Hemoglobin (mean at 10 to 24 wk)	1.905	0.698	0.221 (*p* = 0.0071)
**Anti-diphtheria IgG at 18 months:** ***R***^**2**^ **=** **0.165**			
Sex	0.030	0.052	0.041
Birthweight	0.906	0.414	0.169 (*p* = 0.0300)
Anti-diphtheria IgG in cord blood	−0.103	0.048	−0.151 (*p* = 0.0322)
Anti-diphtheria IgG at age 10 wk	0.223	0.050	0.315 (*p* < 0.0001)
Height-for-age z-score (mean at 10 to 24 wk)	0.016	0.022	0.054
Hemoglobin (mean at 10 to 24 wk)	1.488	0.624	0.172 (*p* = 0.0182)
		**Odds ratio**	**95% CI**
**Seronegative at 18 months:** ***R***^**2**^ **=** **0.243**			
Sex		0.7	0.331, 1.456
Birthweight		1.0	0.997, 0.999 (*p* = 0.0007)
Anti-diphtheria IgG in cord blood		1.5	0.564, 3.991
Anti-diphtheria IgG at age 10 wk		0.01	0.001, 0.143 (*p* = 0.0005)
Height-for-age z-score (mean at 10 to 24 wk)		1.0	0.744, 1.385
Hemoglobin <9.5 g/dl (mean at 10 to 24 wk)		2.1	1.005, 4.573 (*p* = 0.0484)
**PERTUSSIS**			
	**B**	**Standard error of B**	**Standardized** **β**
**Anti-toxin IgG at 24 weeks:** ***R***^**2**^ **=** **0.336**			
Sex	−0.171	0.133	−0.112
Birthweight	−0.098	1.138	−0.009
Anti-toxin IgG in cord blood	−0.705	0.116	−0.527 (*p* < 0.0001)
Weight-for-age z-score (mean at 10 to 24 wk)	0.044	0.072	0.066
Hemoglobin (mean at 10 to 24 wk)	3.052	1.417	0.187 (*p* = 0.0339)
**Anti-toxin IgG at 18 months:** ***R***^**2**^ **=** **0.313**			
Sex	−0.159	0.112	−0.105
Birthweight	1.547	0.950	0.143
Anti-toxin IgG in cord blood	−0.625	0.096	−0.487 (*p* < 0.0001)
Weight-for-age z-score (mean at 10 to 24 wk)	−0.006	0.059	−0.009
Hemoglobin (mean at 10 to 24)	2.474	1.167	0.156 (*p* = 0.0360)
**Anti-Filamentous hemagglutinin IgG at 24 weeks:** ***R***^**2**^ **=** **0.055**			
Sex	−0.078	0.094	−0.086
Birthweight	0.181	0.802	0.029
Anti-filamentous hemagglutinin IgG in cord blood	−0.052	0.090	−0.060
Weight-for-age z-score (mean at 10 to 24 wk)	−0.024	0.051	−0.061
Hemoglobin (mean at 10 to 24 wk)	2.056	0.998	0.213 (*p* = 0.0423)
**PNEUMOCOCCUS 19**			
	**B**	**Standard error of B**	**Standardized** **β**
**Anti-pneumococcus 19 IgG at 18 months:** ***R***^**2**^ **=** **0.091**			
Sex	0.020	0.084	0.018
Birthweight	1.278	0.679	0.151
Anti-pneumococcus 19 in cord blood	0.001	0.058	0.002
Anti-pneumococcus 19 at age 10 wk	0.162	0.067	0.183 (*p* = 0.0165)
Height-for-age z-score (mean at 10 to 24 wk)	−0.011	0.037	−0.023
Hemoglobin (mean at 10 to 24 wk)	2.562	1.019	0.188 (*p* = 0.0129)
		**Odds ratio**	**95% CI**
**Seronegative at 18 months:** ***R***^**2**^ **=** **0.109**			
Sex		0.9	0.456, 1.668
Birthweight		1.0	0.999, 1.000
Anti-pneumococcus 19 in cord blood		1.1	0.997, 1.266
Anti-pneumococcus 19 at age 10 wk		0.7	0.490, 1.041
Height-for-age z-score (mean at 10 to 24 wk)		1.0	0.763, 1.370
Hemoglobin <9.5g/dl (mean at 10 to 24 wk)		2.2	1.136, 4.404 (*p* = 0.0199)
**MEASLES**			
		**Odds ratio**	**95% CI**
**Seronegative at 12 months:** ***R***^**2**^ **=** **0.122**			
Sex		0.8	0.144, 4.896
Birthweight		1.0	0.998, 1.002
Anti-measles IgG in cord blood		1.1	0.770, 1.516
Height-for-age z-score (mean at 10 to 24 wk)		1.0	0.481, 1.966
Hemoglobin <9.5 g/dl (mean at 10 to 24 wk)		5.1	0.831, 31.756 (*p* = 0.0783)

*Analyzed using linear and logistic regression analyses*.

**Table 4:** Associations between serum transferrin receptor and anti-diphtheria, anti-pneumococcus serotype 19 and anti-polio serotype 2 serum IgG concentrations and/or seroconversion, in the birth cohort of Kenyan infants.

**DIPHTHERIA**			
**Seronegative at 18 months: *R*^**2**^ = 0.322**			
		**Odds ratio**	**95% CI**
Sex		1.5	0.534, 3.969
Birthweight		1.0	0.996, 1.000 (*p* = 0.0197)
Anti-diphtheria IgG in cord blood		1.0	0.283, 3.412
Anti-diphtheria IgG at age 10 wk		0.002	0.000, 0.097 (*p* = 0.0016)
Weight-for-age z-score (mean at 10 to 24 wk)		1.4	0.798, 2.369
Serum transferrin receptor at 24 wk		1.2	1.011, 1.315 (*p* = 0.0334)
α-glycoprotein at 24 wk		2.2	0.983, 5.127
Retinol binding protein at age 24 wk		4.2	0.432, 39.888
**PNEUMOCOCCUS SEROTYPE 19**			
	**B**	**Standard error of B**	**Standardized** **β**
**Anti-pneumococcus 19 IgG at 18 months:** ***R***^**2**^ **=** **0.130**			
Sex	−0.127	0.120	−0.110
Birthweight	0.578	1.022	0.063
Anti-pneumococcus 19 in cord blood	−0.011	0.078	−0.014
Anti-pneumococcus 19 at age 10 wk	0.242	0.094	0.273 (*p* = 0.0120)
Height-for-age z-score (mean at 10 to 24 wk)	−0.002	0.051	−0.004
Serum transferrin receptor at 24 wk	−0.908	0.423	−0.247 (*p* = 0.0344)
α-glycoprotein at 24 wk	0.195	0.214	0.093
Retinol binding protein at age 24 wk	0.744	0.456	0.170
		**Odds ratio**	**95% CI**
**Seronegative at 18 months:** ***R***^**2**^ **=** **0.146**			
Sex		1.9	0.754, 4.878
Birthweight		1.0	0.999, 1.001
Anti-pneumococcus 19 in cord blood		1.1	0.974, 1.296
Anti-pneumococcus 19 at age 10 wk		0.6	0.279, 1.153
Height-for-age z-score (mean at 10 to 24 wk)		0.9	0.633, 1.346
Serum transferrin receptor at 24 wk		1.2	1.013, 1.342 (*p* = 0.0327)
α-glycoprotein at 24 wk		0.8	0.362, 1.737
Retinol binding protein at age 24 wk		0.2	0.024. 1.781
**POLIOMYELITIS SEROTYPE 2**			
	**B**	**Standard error of B**	**Standardized** **β**
**Anti-polio 2 IgG at 24 weeks:** ***R***^**2**^ **=** **0.103**			
Sex	0.046	0.091	0.056
Birthweight	−0.037	0.838	−0.006
Anti-polio S2 in cord blood	−0.130	0.096	−0.148
Weight-for-age z-score (mean at 10 to 24 wk)	0.030	0.044	0.092
Serum transferrin receptor at 24 wk	−0.518	0.284	−0.227 (*p* = 0.0719)
α-glycoprotein at 24 wk	−0.091	0.171	−0.063
Retinol binding protein at age 24 wk	0.036	0.305	0.013

*Analyzed using linear and logistic regression analyses*.

### Predictors of Anti-pertussis Serum IgG Concentrations and Seroconversion

For pertussis, we assessed response by measuring antibodies against three antigens: pertussis toxin, FHA and pertactin. Controlling for sex, birthweight, anthropometrics and maternal antibodies, Hb at time of vaccination was the strongest positive predictor of anti-pertussis toxin IgG (*p* = 0.0339) and anti-FHA IgG (0.0423) at 24 wk and the strongest positive predictor of anti-pertussis toxin IgG (*p* = 0.0360) at 18 mo (Table 3). Hb, anemia and TfR were not significant predictors of anti-pertactin IgG, or of response to the Hib and tetanus vaccines.

### Predictors of Anti-pneumococcus Serum IgG Concentrations and Seroconversion

For PnPs, controlling for sex, birthweight, anthropometrics, and maternal antibodies, Hb at time of vaccination was the strongest positive predictor of anti-PnPs 19 serum IgG at 18 mo (*p* = 0.0129) (Table 3). Moderate or severe anemia was the strongest risk factor for seronegativity at 18 mo: anemic infants had 2.2 times the risk of being unprotected against PnPs 19 at 18 mo (*p* = 0.0199). Controlling for sex, birthweight, anthropometrics, inflammation, vitamin A status and maternal antibodies, TfR at 24 wk was the strongest negative predictor of anti-PnPs 19 IgG at 18 mo (*p* = 0.0344) (Table 4). Controlling for the same factors, TfR was the strongest risk factor for seronegativity against PnPs 19 at 18 mo (*p* = 0.0327). Hb, anemia and TfR were not significant predictors of response to the other PnPs serotypes.

### Predictors of Anti-polio Serum IgG Concentrations and Seroconversion

Controlling for sex, birthweight, anthropometrics, inflammation, vitamin A status and maternal antibodies, TfR at 24 wk was the strongest negative predictor of anti-polio S2 IgG at 24 wk (*p* = 0.0719) (Table 4). Hb, anemia and TfR were not significant predictors of response or seroconversion to polio S1 and S3.

### Predictors of Anti-measles Serum IgG Concentrations and Seroconversion

For measles, moderate or severe anemia was the strongest risk factor for seronegativity at 12 mo: anemic infants had 5.1 times the risk of being unprotected against measles at 12 mo (*p* = 0.0783) (Table 3). Hb and anemia were not significant predictors of response to MV at age 18 mo.

### Randomized Trial Cohort Follow-Up Study

The randomized trial began recruiting in October 2014 and follow up was to March 2019. Comparing the two groups that received the 5 mg daily dose of iron (the iron only and the iron and GOS groups), there were no significant differences in any of the measured parameters at age 11.5 mo or at 4.5 y (data not shown). Notably, there were no significant differences in anti-measles serum IgG, seroconversion or avidity comparing the iron only and iron and GOS groups at any time point. Therefore, we pooled the iron only and the iron and GOS groups into a single iron group that had received 5 mg of iron daily for 4 mo, and compared this new iron group to the no-iron group as control (Figure 2). 127 infants were assessed at age 11.5 mo (85 infants in the iron group and 42 infants in the control group) and 88 were assessed at age 4.5 y. Median (IQR) birthweight was higher in the control group than in the iron group (3,300 (3,000–3,500)g vs. 3,000 (2,600–3,500)g) (*p* = 0.0299). Nine (10.5%) infants in the iron group and four (10.0%) infants in the control group were LBW (<2,500 g). Table 5 shows anthropometrics, Hb, iron and inflammatory parameters and anti-measles serum IgG concentrations at age 7.5 mo, 11.5 mo and 4.5 y. There were no significant group differences in any of the parameters at 7.5 mo. During the intervention, there was a marked improvement in iron status in the iron group, but not in the control group: there were significant group effects for Hb (*p* = 0.0001) and plasma ferritin (PF, *p* = 0.0198), with higher Hb and PF in the iron group compared to the control group and there were significant group by time interactions for Hb (*p* = 0.0001), PF (*p* = 0.0001) and TfR (*p* < 0.0001).

**Table 5:** In a randomized trial follow-up study, age, anthropometrics, biomarkers of iron, vitamin A and inflammation, and anti-measles serum IgG concentrations in Kenyan infants receiving 5 mg iron daily or no-iron control for 4 months, and given measles vaccine at age 9 mo and 18 mo, measured at baseline (age 7.5 mo), end of intervention (age 11.5 mo) and follow-up (age 4.5 y).

	**Baseline of intervention**	**End of intervention**	**Follow up**	***p*****-values**		
				**Group**	**Time**	Group*time
*n*				-	-	-
Iron	85	85	55			
Control	42	42	33			
Age, mo^a^				-	-	-
Iron	7.4 (7.1 – 8.5)	11.1 (10.8 – 12.4)	54.9 (52.3 – 56.7)			
Control	7.5 (7.0 – 8.6)	11.3 (10.7 – 12.6)	53.9 (51.8 – 56.7)			
Weight-for-age z-score^b^				0.3745	0.9005	0.5555
Iron	−0.52 ± 1.17	−0.50 ± 1.21	−1.00 ± 0.86			
Control	−0.30 ± 1.03	−0.34 ± 1.18	−1.03 ± 0.76			
Weight-for-height z-score				0.0954	0.0181	0.8032
Iron	−0.34 (−1.10 – 0.19)	−0.32 (−0.94 – 0.61)	−0.72 (−1.15 −0.22)			
Control	−0.15 (−0.89 – 0.92)	0.29 (−0.84 – 0.83)	−0.55 (−1.07 −0.13)			
Height-for-age z-score				0.6351	<0.0001	0.9142
Iron	−0.26 ± 1.19	−0.66 ± 1.34	−0.91 ± 0.96			
Control	−0.35 ± 1.04	−0.77 ± 1.02	−0.97 ± 0.84			
Hemoglobin, g/dl				0.0001	0.0131	0.0001
Iron	10.4 (9.5 – 10.9)	11.1 (10.6 – 12.0)	11.3 (10.5 – 12.0)			
Control	10.3 (9.4 – 10.7)	9.8 (9.2 – 10.7)	11.2 (10.6 – 11.9)			
Plasma ferritin, μg/L				0.0198	0.2427	0.0001
Iron	15.5 (10.0 – 26.8)	23.6 (16.8 – 35.0)	25.8 (17.7 – 39.0)			
Control	15.3 (8.1 – 32.2)	11.7 (8.1 – 27.8)	20.0 (10.3 – 29.7)			
Transferrin receptor, mg/L				0.1033	0.2224	<0.0001
Iron	12.4 (8.7 – 15.8)	8.8 (7.9 – 11.5)	7.01 (6.0 – 9.5)			
Control	10.3 (8.4 – 14.7)	12.7 (10.1 – 18.1)	7.6 (6.3 – 9.0)			
Retinol binding protein, μmol/L				0.1632	0.0160	0.6440
Iron	0.94 (0.80 – 1.04)	0.95 (0.81 – 1.22)	0.84 (0.64 – 0.94)			
Control	0.90 (0.73 – 1.05)	0.94 (0.77 – 1.08)	0.73 (0.61 – 0.85)			
C-reactive protein, mg/L				0.7476	0.7605	0.2592
Iron	1.10 (0.50 – 4.20)	1.60 (0.50 – 5.05)	0.17 (0.01 – 1.58)			
Control	1.20 (0.55 – 7.45)	1.10 (0.38 – 4.30)	0.27 (0.06 – 1.70)			
α-glycoprotein, g/L				0.8907	0.1656	0.3847
Iron	0.93 (0.68 – 1.67)	1.23 (0.75 – 2.03)	0.63 (0.47 – 0.80)			
Control	1.02 (0.69 – 2.08)	1.09 (0.70 – 2.18)	0.70 (0.58 – 1.21)			
Measles IgG, IU/ml				0.0415	<0.0001	0.2952
Iron	0.01 (0.01 – 0.03)	0.49 (0.23 – 0.72)	0.37 (0.19 – 0.78)			
Control	0.01 (0.01 – 0.03)	0.39 (0.11 – 0.73)	0.33 (0.25 – 0.84)			

a *All such data as medians (IQR)*.

b *All such data as means ±SD. Analyzed using linear mixed effect model (LMM) analyses with Bonferroni correction on baseline and end of intervention parameters. For anti-measles serum IgG the LMM analysis on the primary vaccine response is shown*.

### Anti-measles Serum IgG Concentrations

**Figure 4:**
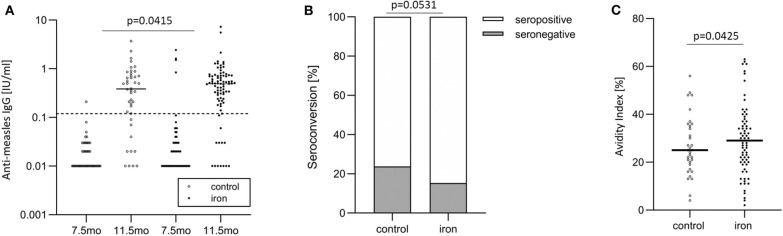
In the randomized controlled trial follow-up study, anti-measles serum IgG concentrations, seroconversion, and IgG avidity in the control and iron groups. **(A)** Anti-measles serum IgG concentrations at age 7.5 and 11.5 months (at baseline and end of intervention), **(B)** seroconversion at age 11.5 months (at end of intervention), and **(C)** IgG avidity at age 11.5 months (at end of intervention).

For primary vaccine response (age 11.5 mo), there was a significant group effect (*p* = 0.0415) with higher anti-MV serum IgG in the iron group and a significant time effect (7.5 vs. 11.5 mo; *p* < 0.0001), but no group^*^time interaction (*p* = 0.2952) (Table 5 and Figure 4A). For secondary vaccine response, there was a significant time effect (11.5 mo vs. 4.5 y; *p* < 0.0001), but no group effect (*p* = 0.6800) and no group^*^time interaction (*p* = 0.6445).

### Seroconversion and Anti-measles IgG Avidity

Before vaccination (at age 7.5 mo), 95.3 vs. 97.6% of infants were seronegative against MV in the iron and control group, respectively. After vaccination, at age 11.5 mo, 15.3 vs. 23.8% of infants were seronegative against MV in the iron and control group (Figure 4B). At age 4.5 y, 9.1 vs. 12.1% of infants were seronegative against MV in the iron and control group. Controlling for WHZ, IDA, RBP and age, being in the control group was the strongest risk factor for seronegativity at age 11.5 mo: infants in the control group had 3.1 times greater risk of being unprotected against measles at this age (*p* = 0.0531) (Table 6). At age 11.5 mo and 4.5 y, in the iron and control groups, mean (±SD) avidity was 29.1 ± 14.3 vs. 26.8 ± 12.5% (Figure 4C) and 69.1 ± 14.2 vs. 65.6 ± 14.9%, respectively. Controlling for the same parameters as above, being in the control group was the strongest risk factor for low avidity at age 11.5 mo: infants in the control group had 3.2 times greater risk of having a low avidity at this age (*p* = 0.0425) (Table 6). In the same models, there was no significant group effect on seroconversion or avidity at age 4.5 y.

**Table 6:** In a randomized trial follow-up study, predictors of seroconversion and antibody avidity in Kenyan infants receiving 5 mg iron daily or no-iron control for 4 months, and given measles vaccine at age 9 mo, measured end of intervention (age 11.5 mo).

	**Odds ratio**	**95% CI**
**Seronegative at end of intervention:** ***R***^**2**^ **=** **0.211**		
Control group	3.1	0.985, 9.729 (*p* = 0.0531)
Iron deficiency anemia	2.9	0.918, 9.358
Weight-for-height z-score	0.6	0.416, 0.963
Age	0.4	0.203, 0.921
Retinol binding protein	0.2	0.030, 1.334
**Low avidity at end of intervention:** ***R***^**2**^ **=** **0.264**		
Control group	3.2	1.040, 9.647 (*p* = 0.0425)
Iron deficiency anemia	1.1	0.383, 2.925
Weight-for-height z-score	0.7	0.518, 1.081
Age	0.3	0.183, 0.600 (*p* = 0.0003)
Retinol binding protein	1.5	0.251, 8.732

*Analyzed using logistic regression analyses*.

## Discussion

Our main findings are, in Kenyan infants: (1) ID and anemia were common at time of vaccination: over 50% of infants were already anemic at age 10 and 14 wk, and 57% had IDA at 24 wk; (2) controlling for most established risk factors affecting vaccine response: (a) Hb at time of vaccination was the strongest positive predictor of anti-diphtheria IgG and anti-pertussis toxin IgG at 24 wk (primary response) and 18 mo (secondary response), and of anti-PnPs 19 IgG at 18 mo; (b) moderate to severe anemia at the time of vaccination more than doubled the risk of being seronegative against diphtheria and PnPs 19 at 18 mo; (c) TfR at 24 wk was a strong negative predictor of seroconversion against diphtheria and PnPs 19 at 18 mo; and (3) iron supplementation of infants at time of measles vaccination at age 9 mo improved the primary vaccine response measured at age 11.5 mo, as indicated by higher anti-measles serum IgG concentrations, seroconversion and IgG avidity.

In the birth cohort study, more than half of infants were already anemic at age 10 wk (Table 1). Term infants born to iron-sufficient mothers should have adequate birth iron stores to cover iron requirements for the first 4 to 6 months (26). However, in Sub-Saharan Africa, 46% of pregnant women have IDA (27), reducing maternal-fetal iron transfer (26), the umbilical cord is often clamped too early, and 15–25% of newborns have LBW (28). These sharply reduce newborn iron stores: it is estimated that body iron is 40–50% lower in newborns who are LBW and/or whose mothers were anemic during pregnancy (26). Low iron stores at birth together with frequent infections increasing serum hepcidin (29), and diarrhea/intestinal parasites causing blood loss, result in many infants depleting their iron stores within 3–4 months after birth. Although representative studies in early infancy are lacking, data from our study and others suggest anemia affects many infants in LMIC at the time of vaccination (7, 8).

Defining iron status in infants at age <24 wk is difficult (26, 30). There are major shifts of iron from fetal Hb to splenic macrophages and hepatocytes and then to a rapidly increasing red cell mass (26, 30). In African infants, common infections increase SF (an acute phase protein) and elevate serum hepcidin, which causes anemia of inflammation (29). TfR levels are also highly variable, with lower values in the first 2 months, and gradually rising values thereafter (30). These patterns were visible in our infants, who despite having low Hb values and high rates of anemia, showed high SF and low TfR concentrations at 10 and 14 wk, but low SF and high TfR concentrations at 24 wk (Table 1). Because there are no consensus normative values for iron biomarkers at age <24 wk (30), we cannot be certain that ID explains the link between anemia with vaccine response at age <24 wk. However, it is likely that most of the early anemia was due to ID, and at 24 wk, when we could begin to apply biomarkers to define iron status, TfR (a measure of iron deficient erythropoiesis) was a predictor of vaccine response (Table 4).

Previous reviews (5, 9, 31) have suggested multiple mechanisms by which iron status might influence adaptive immunity. In some animal and cell models, ID reduces the proportion of mature T-cells and impairs T-cell activation and proliferation, although in many cell models iron chelators have been used (5, 9, 31). Some studies have also shown detrimental effects of ID on B-cell numbers or function, but others have not (5, 9–11, 31). In humans, studies on ID and immune function show varying results depending on what aspect of immunity is measured and the severity of ID, age and/or underlying nutritional status (5, 9). Iron uptake via TfR1 is essential for lymphocyte development, and clinically, a homozygous mutation in TfR1 causes severe immunodeficiency in children and reduced numbers of circulating memory B cells (11). *In vitro* this mutation prevented T- and B-cell proliferation and addition of high concentrations of iron citrate *in vitro* rescued the proliferative defect (11). Thus, adequate iron availability may be critical for adaptive immunity. If ID critically limits iron availability to responding lymphocytes (10), this may explain our findings that ID and anemia during early infancy are associated with an impaired response to vaccination. If ID limits the development of memory B-cells (10) it may reduce long-term immunity (32). Recent studies showing impaired antigen-specific immune responses in hypoferremic mice and lower antibody concentrations in patients with iron-refractory IDA support this concept (Joe Frost et al., personal communication 2020).

We are aware of no controlled intervention studies examining the effect of iron supplementation on infant vaccine response in Africa. Most previous studies on iron status and vaccines have been small and cross-sectional, and have produced equivocal results (5). In South Africa, the response to a diphtheria toxoid vaccine was measured in children with IDA, milder ID and non-anemic controls; the numbers with a positive response was 12 of 14 in the controls, 2 of 11 in the anemic children and 0 of 7 in those with ID (33). An observational study in eight LMIC assessing links between enteropathogen infection, undernutrition, and child health (*n* = 1449) found borderline significant associations between TfR and oral polio vaccine response at age 7 and 15 mo (34). In a cross-sectional study of Ecuadorian children (*n* = 1,162) who had previously received diphtheria and tetanus vaccines, anemic children older than age 1 y had lower antibody levels than controls and more anemic children were seronegative for diphtheria compared with controls (12.5 vs. 18.1%, respectively) (35).

Because different vaccine types induce different immune responses (36), they may be differentially affected by ID. ID may have greater detrimental effects on T cell numbers and function, rather than on B cells (5), although not all studies agree (10). This may help explain why in our study, anemia predicted response to diphtheria, pertussis and measles vaccine, but not to Hib and most serotypes of PnPs. Measles, a live vaccine, and the protein antigens of diphtheria and pertussis, induce a strong T-dependent response, including induction of antigen-specific Tfh cells and germinal centers producing memory B cells, and these processes are highly iron dependent (10). Unless effectively conjugated with an adjuvant protein, bacterial polysaccharide antigens (e.g., Hib, PnPs) induce primarily T-independent responses and do not generate Tfh cells or germinal centers (37); thus, their immunogenicity, even when conjugated, might be less affected by iron availability.

In the randomized trial, our findings suggest that iron supplementation of mostly IDA infants at time of MV improves the primary response to the vaccine. Nearly all measles deaths globally occur in young children in LMIC countries (38). The Kenyan MoH recommends MV at age 9 mo because measles infection occurs frequently in infancy; however, primary vaccination failures occur in up to 10–15% of infants vaccinated at age 9 mo (38). In the WHO Africa region, median effectiveness of the first MV given at age 9–11 months was 73%, but the range was from 26 to 95%, showing more variability and lower effectiveness than all other WHO regions (39). The development of a high avidity antibody response is critical for protection against measles virus (38) and avidity to MV is generally lower in children vaccinated at 9 mo compared to later ages (38). Our findings suggest IDA may contribute to primary MV failure by reducing both antibody quantity and quality.

The strengths of the birth cohort study include a large sample size and an 18-month prospective design. All infants received three doses (four for OPV) of the identical vaccine administered and recorded by the study team, improving reliability. Hb and anthropometric indices were measured at three vaccination time points during early infancy, and responses to multiple vaccines were measured at multiple time points, which likely minimized misclassification bias and sharpened case definition. We tried to minimize confounding by controlling for most established risk factors affecting vaccine response; however, our results may in part reflect residual confounding not accounted for in the models. In many previous studies linking nutrition to vaccine response, seroprotective levels were reached in nearly all children irrespective of nutritional status (3). In our study, the response to vaccination was variable; for example, only 68% of infants in the birth cohort study had protective anti-diphtheria serum IgG concentrations at age 18 mo, increasing our ability to detect associations.

Our study also has limitations. We did not specifically control for breastfeeding, but in the study area nearly all mothers partially or fully breastfeed during the first 6 mo. Also, we did not control for chronic parasitic infections, but their prevalence is <2% in early infancy in this area (40). Because of the difficulties of defining iron status before age 6 mo, we cannot be sure that ID is the major cause of the anemia in our birth cohort. However, it is likely to be the major cause and thus anemia *per se* may not be a predictor of vaccine response, but rather only a surrogate for ID. As our main outcomes, we assessed antibody concentrations and seroprotection, but these are only surrogates of disease protection induced by vaccination. The Bacillus-Calmette-Guérin (BCG) vaccine may provide protection against other infectious diseases than tuberculosis (41) but we did not record BCG administration nor measure BCG vaccine response. In the randomized trial, we compared the MV response in infants receiving a MNP with iron to infants receiving the same MNP without iron. It is possible that components of the MNP other than iron (e.g., vitamin A, zinc) could have had an impact on MV response; however, reviews suggest the only clear benefit of MNPs is an improvement in iron status and hemoglobin (42). We also did not measure parameters of cellular immune responses, such as T cell proliferation, cytokine secretion or delayed-type hypersensitivity tests, which could have provided additional information of the immunogenicity of the vaccines.

To our knowledge these are the first prospective data from Africa assessing the impact of anemia and ID at the time of vaccination on response to a range of pediatric vaccines. Our data suggest that anemia/ID at the time of infant vaccination may impair the response to diphtheria and pertussis vaccines and that improving iron status may improve response to measles vaccine. Clearly, our findings need to be confirmed in other prospective cohorts and/or by larger intervention trials. Future research should assess the potential impact of anemia/ID on response to oral vaccines, such as rotavirus vaccine. Powerful emerging techniques combining mass cytometry and systems-level-omics tools may allow identification of the mechanisms underlying the effects of iron status on the infant immune system and response to vaccines. If confirmed, our findings argue strongly for early detection and correction of IDA in infancy. Because anemia is so common in African infants and because the vaccine-preventable disease burden is so high (1, 2), even if IDA only modestly reduces immunogenicity of childhood vaccines its prevention could have major benefits.

## Data Availability Statement

The raw data supporting the conclusions of this article will be made available by the authors, without undue reservation.

## Ethics Statement

The studies involving human participants were reviewed and approved by Study 1: Institutional Review Boards at Kenyatta National Hospital (KNH), Case Western Reserve University and the Stanford University School of Medicine. Study 2: Institutional Review Boards of the KNH/University of Nairobi (KNH/UoN) and the Zurich Cantonal Ethical Commission. Approval for the follow-up study was given by (KNH/UoN). Written informed consent to participate in this study was provided by the participants' legal guardian/next of kin.

## Author Contributions

FK, IM, AL, and CK conceived and conducted the birth cohort study. NS, MU, EM, DP, SK, FK, and MZ conceived and conducted the randomized trial. All authors provided inputs on the data analyses. NS and MZ wrote the first draft of the manuscript. All authors contributed to the editing and the finalization of the manuscript.

## Conflict of Interest

The authors declare that the research was conducted in the absence of any commercial or financial relationships that could be construed as a potential conflict of interest.
